# MicroRNA Profiling in Intraocular Medulloepitheliomas

**DOI:** 10.1371/journal.pone.0121706

**Published:** 2015-03-25

**Authors:** Deepak P. Edward, Hind Alkatan, Qundeel Rafiq, Charles Eberhart, Saleh Al Mesfer, Nicola Ghazi, Leen Al Safieh, Altaf A. Kondkar, Khaled K. Abu Amero

**Affiliations:** 1 King Khaled Eye Specialist Hospital, Riyadh, Saudi Arabia; 2 Wilmer Eye Institute, John Hopkins University School Of Medicine, Baltimore, MD, United States of America; 3 Department of Ophthalmology, College of Medicine, King Saud University, Riyadh, Saudi Arabia; 4 Department of Ophthalmology, College of Medicine, University of Florida, Jacksonville, FL, United States of America; University of Connecticut Health Center, UNITED STATES

## Abstract

**Purpose:**

To study the differential expression of microRNA (miRNA) profiles between intraocular medulloepithelioma (ME) and normal control tissue (CT).

**Material and Methods:**

Total RNA was extracted from formalin fixed paraffin embedded (FFPE) intraocular ME (n=7) and from age matched ciliary body controls (n=8). The clinical history and phenotype was recorded. MiRNA profiles were determined using the Affymetrix GeneChip miRNA Arrays analyzed using expression console 1.3 software. Validation of significantly dysregulated miRNA was confimed by quantitaive real-time PCR. The web-based DNA Intelligent Analysis (DIANA)-miRPath v2.0 was used to perform enrichment analysis of differentially expressed (DE) miRNA gene targets in Kyoto Encyclopedia of Genes and Genomes (KEGG) pathway.

**Results:**

The pathologic evaluation revealed one benign (benign non-teratoid, n=1) and six malignant tumors (malignant teratoid, n=2; malignant non-teratoid, n = 4). A total of 88 miRNAs were upregulated and 43 miRNAs were downregulated significantly (P<0.05) in the tumor specimens. Many of these significantly dysregulated miRNAs were known to play various roles in carcinogenesis and tumor behavior. RT-PCR validated three significantly upregulated miRNAs and three significantly downregulated miRNAs namely miR-217, miR-216a, miR-216b, miR-146a, miR-509-3p and miR-211. Many DE miRNAs that were significant in ME tumors showed dysregulation in retinoblastoma, glioblastoma, and precursor, normal and reactive human cartilage. Enriched pathway analysis suggested a significant association of upregulated miRNAs with 15 pathways involved in prion disease and several types of cancer. The pathways involving significantly downregulated miRNAs included the toll-like receptor (TLR) (p<4.36E-16) and Nuclear Factor kappa B (NF-κB) signaling pathways (p<9.00E-06).

**Conclusions:**

We report significantly dysregulated miRNAs in intraocular ME tumors, which exhibited abnormal profiles in other cancers as well such as retinoblastoma and glioblastoma. Pathway analysis of all dysregulated miRNAs shared commonalities with other cancer pathways.

## Introduction

MiRNAs are short (approximately 22 nt), endogenous, non-coding, single-stranded RNA regulatory molecules that regulate gene expression post-transcriptionally by degrading messenger RNA (mRNA) targets and/or by blocking their translation [1, 2]. Because of their unique post-transcription and protein-translation regulatory functions, miRNAs are known to regulate several key cellular and biological processes including tissue differentiation, development, growth, proliferation, and apoptosis [3]. MiRNAs regulate 30% to 90% of protein-coding human genes, thus seem to have the potential to modulate complex physiological or disease phenotypes. MiRNAs have been reportedly expressed in tissues, whole blood, serum plasma, and other body fluids [4]. Unlike mRNA, they are present in a stable form that is protected from endogenous RNase activity [5]. These features make miRNAs extremely attractive for genetic epidemiological research, where archived FFPE tissue, blood, or other biological fluids are most often available. In addition, miRNA expression profiles seem to correlate well between fresh and FFPE samples [6]. MiRNA dysregulation has been implicated in the development and progression of various pathological conditions including cancer [7, 8] and is emerging as a potential biomarker in several diseases [9].

Intraocular medulloepithelioma is an uncommon congenital tumor of the undifferentiated, non-pigmented ciliary epithelial body, rarely arising from the iris, optic nerve head, or retina [10–12]. The tumor is predominantly diagnosed in children at the median age of 2 to 5 years [13, 14]. Although the tumor is rare as compared to retinoblastoma, it is still the second most common primary intraocular neoplasm. Most patients with medulloepithelioma commonly present with visual symptoms, pain, protusion of the eye or ciliary body cystic mass, cataract, glaucoma, lens coloboma, and leukocoria. The ciliary body mass with cysts within the tumor is a classic feature of medulloepithelioma [11, 13, 15]. Ultrasonographic examination is ideal to analyze the anterior segment [16] and radiological investigations can be especially beneficial if the ciliary body mass does not involve the retina [17]. Histologically, the tumor resembles the medullary epithelium of the embryonic neural tube or primitive retina. The pathologic findings are similar to another rare tumor of the central nervous system (CNS) i.e. medulloepithelioma of the CNS, but exhibits a different clinical behavior [18]. Ocular medullopeitheliomas can be classified as benign or malignant [13]. Immunohistochemical markers have different patterns of reactivity depending on whether the tissue of interest is neuroepithelial or heteroplastic. Overall, the survival rate of patients with intraocular medulloepithelioma is excellent. In localized tumors, the eyes can often be salvaged by tumor resection. Malignant intraocular medulloepitheliomas can cause significant ocular morbidity and extraocular extension, thus requiring enucleation or exenteration for treatment.

Identification of miRNAs in ocular cells [19], aqueous humor [20], and vitreous fluid [21] suggests that miRNAs may have roles to play in the development and function of the eye and eye diseases. Many other studies have also reported miRNA dysregulation in several ocular diseases. These studies included differential miRNA expression in central epithelium of transparent and cataractous lenses [22] with overexpression of let-7b in lenses with greater opacity [23]; downregulation of miR-29b increasing fibrosis risk in Tenon’s fibroblasts after glaucoma filtering surgery [24]; miR-24 blocking p53 tumor surveillance contributing to retinoblastoma [25]; and downregulation of miR-146a [26] and miR-200b [27] in retinal endothelial cells in diabetics.

In recent years, many studies have shown that there are alterations in miRNA profiles in tumor tissue when compared with normal tissue. Some reports noted a general downregulation of miRNAs in cancerous tissue suggesting that these non-coding RNAs may act as tumor suppressors. MiRNAs dysregulation can drive or antagonize tumorigenesis at various steps that include miRNA biogenesis, post-transcriptional miRNA changes, and alterations in RNA sequences [28].

Based on the literature review, we hypothesized that the study of differential expression of miRNA profiles between medulloepithelioma tissues and normal control would give us clues about the role of miRNA dysregulation in the pathogenesis of this rare tumor.

## Material and Methods

The clinical history and histological features of the patients with intraocular medulloepithelioma were retrieved from the medical records in a de-identified fashion. The study was approved by the Institutional Review Board at the Johns Hopkins University School of Medicine, USA and at the King Khaled Eye Specialist Hospital, KSA. The institutional review board waived the need for consent.

### Tissue dissection and fixation

The tumor tissues (n = 7) were dissected from the FFPE tissue block using a dissecting microscope with a sterile No. 11 blade. For control tissue (n = 8), the ciliary body and epithelium of enucleated eyes with retinoblastoma, where the tumor was confined to the posterior segment, were dissected.

### Extraction of miRNAs from FFPE tissue

Total RNA including miRNA was extracted from FFPE samples using miRNeasy FFPE kit (Qiagen) following the manufacturer’s instructions. Each sample was deparaffinized with Qiagen’s deparaffinization solution. Lysis buffer and proteinase K were added to release nucleic acid molecules. After the recommended heat treatments, DNase booster buffer and DNase I were added to the supernatant to remove genomic DNA and any small fragments of DNA since the latter are found in FFPE samples after long-term fixation and storage. Followed by the addition of buffer RBC and 100% ethanol, the entire sample volume was run on RNeasy MinElute spin columns, which were washed with buffer RPE twice according to the user manual. Control samples were eluted in 20 μl and ME samples were eluted in 60 μl of RNase free water. RNA concentration was measured using NanoDrop ND 1000. RNA quality was determined with Agilent bioanalyzer RNA 6000 Nano chip. Samples with RNA integrity number of at least 7 were used for the miRNA arrays.

### Affymetrix GeneChip miRNA Arrays

For analysis with Affymetrix GeneChip miRNA Arrays (Affymetrix), we prepared samples with 150 ng of RNA. Samples were labeled with the FlashTag Biotin HSR labeling kit according to the manufacturer’s instructions as follows. A tailing reaction was performed, RNA concentration was adjusted, and RNA Spike Control Oligos were added. ATP diluted in 1 mM Tris for total RNA was mixed with 10X reaction buffer, 25mM MnCl2, and PAP enzyme to make the Poly A Tailing Master Mix in a nuclease-free tube. The Poly A Trailing master mix, RNA sample, and oligo mix were incubated. At this point, the 5X FlashTag Biotin HSR Ligation Mix and T4 DNA Ligase were added to each sample and the ligation mixtures were incubated. Ligation reactions were hybridized with Affymetrix GeneChip miRNA arrays. Hybridization cocktail was added to each biotin-labelled ligation mixture and the resulting mix was applied to an array and incubated overnight. Arrays were then washed and stained with Affymetrix kit and fluidics Station 450 according to protocol FS450_0002 and scanned with Affymetrix Command Console (AGCC) Software. Data was analyzed using expression console 1.3 software and uploaded to the NCBI GEO database (Accession ID: GSE62367).

### MiRNA validation by RT-PCR

Each miRNA sample (12 ng) was reverse transcribed to cDNA utilizing the TaqMan MicroRNA Reverse Transcription Kit (Life Technologies). The product was then pre-amplified with TaqMan PreAmp master mix and pooled Taqman assays. The qPCR mixture was run on the 7900HT fast real-time PCR system (Life Technologies). Each qPCR reaction contained previously amplified template, TaqMan universal master mix II with no UNG and TaqMan miRNA primer assays (Life Technologies). The six different primer assays used were for miR-211, miR-509-3p, miR-146a, miR-217, miR-216a, and miR-216b miRNAs. U6 snRNA was used as the endogenous control.

### Statistical analysis

#### Method for differential profiling

Control (n = 8) and tumor (n = 7) samples were each run on a separate Affymetrix GeneChip miRNA array and data was analyzed with Partek Genomics Suite 6.6 (Partek, Inc, St. Louis, MO). Raw data was processed using Robust Multi-array Analysis (RMA). Quality control was performed by Principle Component Analysis (PCA). Tumor versus control fold change values were calculated. One-way ANOVA was used to calculate p-values. Volcano plots were constructed with TIBCO Spotfire DecisionSite client v9.1.2 with-Log10 (p-value) on the y-axis and log2 (fold change) on the x-axis.

#### Method for miRNA validation

Candidate miRNAs were quantified by the comparative cycle threshold (C_T_) method on an ABI PRISM 7900 HT Sequence Detection System. Real-time PCR reactions for each differentially expressed miRNA and template were done in triplicate. U6 snRNA was used as the endogenous control to normalize the data. The delta C_T_ (ΔC_T_), relative ΔΔC_T,_ and fold change values were calculated in pooled samples. Statistical analysis was done using Mann Whitney U test with significance level of 0.05 and 2-tailed hypothesis.

#### MiRNA-targeted pathway analysis

The web-based DNA Intelligent Analysis (DIANA)-miRPath v2.0 was used (http://www.microrna.gr/miRPathv2) to perform enrichment analysis of differentially expressed miRNA gene targets in Kyoto Encyclopedia of Genes and Genomes (KEGG) pathway v58.1. Following the inclusion of DE miRNAs in the web tool the web server utilizes miRNA targets predicted with high accuracy based on DIANA-microT-CDS and/or experimentally validated transcripts from TarBase v6.0 and provides a p-value for each pathway by applying Fisher’s method [29]. The default settings of the web server includes a score cutoff of 0.8 for the target prediction that predicts around 350 mRNA targets per miRNA, a false discovery rate (FDR) method to correct multiple hypothesis testing, and a p-value threshold of 0.05.

## Results

The clinical data of the patients are summarized in Table 1. Of the seven patients, one had a benign medulloepithelioma and six had malignant medulloepitheliomas.

**Table 1:** Clinical and Demogrpahic details of patients included in the study.

**Case**	Sex/Age at presentation	Main clinical features	Pathologic findings	Pathologic diagnosis	Tumor invasion extraocular/scleral invasion	Outcome
1	M/4y; age at tissue Dx: 9y	VA, OS HM; buphthalmos, glaucoma, IOP: 37mmHg; early cataract	Non-teratoid medulloepithelioma; undifferentiated cells resembling Rb cells; frequent mitotic figures; corneal invasion with staphyloma; optic disc cupping	Malignant non-teratoid; OS	Corneal	Tm: Enucleation; AW-FU period 18y
2	F/13Y; age at tissue Dx:17y	Visible grape like creamy white mass in AC& uveitis IOP:41 mmHg	Non-teratoid medulloepithelioma; rosettes; few mitotic figures; cluster of tumor in AC; Alcian blue positive cystic spaces	Benign non-teratoid; OS	None	Tm: Enucleation; buphthalmic AW-FU period 1y
3	M/6Y; age at tissue Dx:6Y	Visible mass; ACG, IOP 36mmHg; cataract/lens sublaxation, uveitis	Non-teratoid medulloepithelioma; myxoid areas in tumor, lens capsule rupture; frequent mitotic figures; invasive features	Malignant non-teratoid; OD	Iris; corneal	Tm: Enucleation; AW-FU: 11y
4	M/2Y; age at tissue Dx:3Y	Buphthalmos; pseudohypopyon; cataract, corneal edema, iris neovas; glaucoma, IOP:40	Non-teratoid medulloepithelioma; undifferentiated large pleomorphic cells; frequent mitotic figures; Alcian blue positive cystic spaces; few rosettes; staphyloma, break in Descemet’s membrane	Malignant non-teratoid; OS	Corneoscleral tissue; AC angle	Tm: Enucleation; AW-FU period: 10Y
5	F/3Y; age at tissue Dx:5y	Buphthalmos, anterior staphyloma; proptosis; pain & redness; corneal opacity; TRD & exudation	Teratoid medulloepithelioma; neuroepithelial component; fibrillar cytoplasm & positive GFAP cells; moderate mitosis; Alcian blue positive cystic spaces; invasion: cornea/subconjunctival	Malignant teratoid; OD	Extrascleral: subconj nodule; corneal: posterior stroma	Tm: Enucleation; chemo: 6 cycles; AW-FU: 4Y
6	M/1.5Y; age at tissue Dx:2y	History of trauma; shallow AC; inferonasal vascular lesion on iris; distorted pupil; cortical material	Non-teratoid medulloepithelioma; focal necrosis; mitotic figures; involvement of ant segment/iris; focal intrascleral extension	Malignant non-teratoid; OS	Ant segment & iris; intrascleral: focal	Tm: Enucleation; AW-FU period: 1Y
7	F/ 0.5Y; age at tissue Dx:5y	Ciliary body mass seen through pupil	Teratoid medulloepithelioma; TRD; absent iris & lens neural/glandular/cartilagenous elements; corneal perforation/extraocular extension	Malignant teratoid; OS	Extraocular; intrascleral	Tm: Enucleation; AW-FU period: 1Y

M: Male; F: Female; Y: Years; AC: anterior chamber; OD: right eye; OS: left Eye; HM: hand motion; TRD: tractional retinal detachment.

### Affymetrix GeneChip miRNA Arrays Analysis

**Fig 1:**
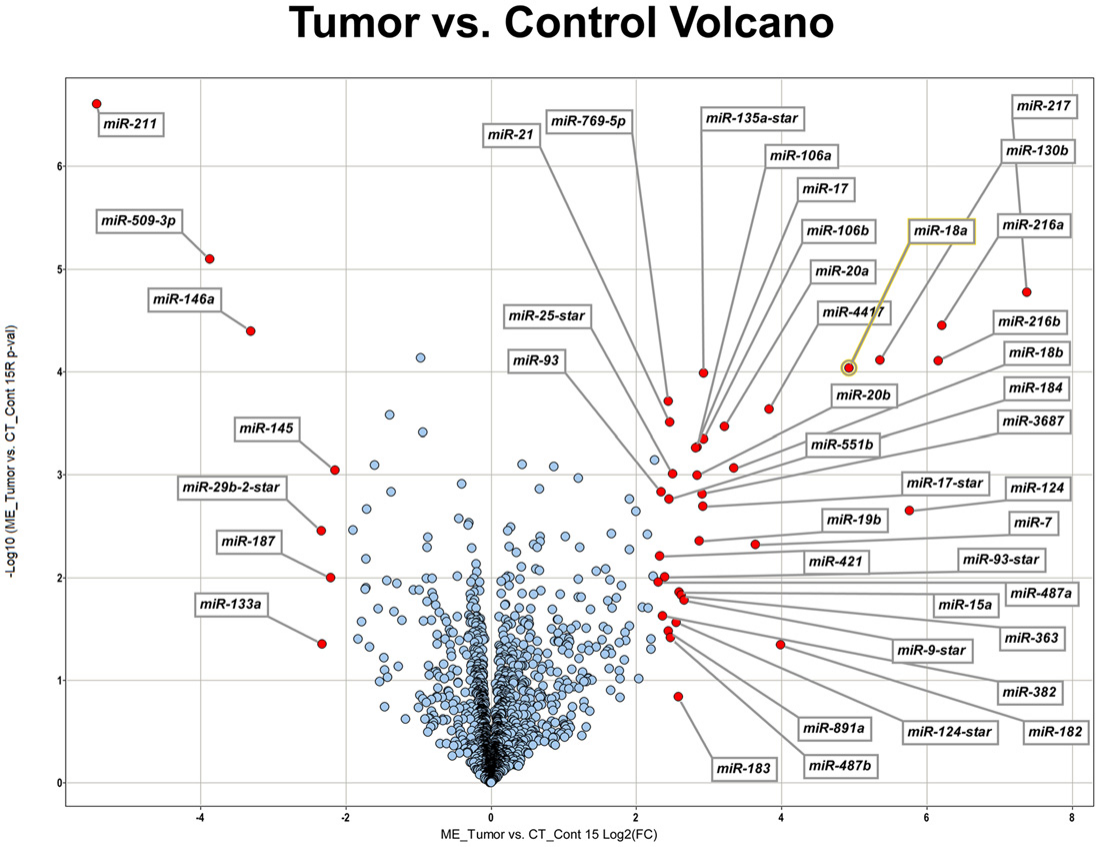
Volcano plot of differentially expressed miRNAs in seven cases of medulloepithelioma. The x-axis shows the log2 fold-change in miRNA expression and y-axis shows the-Log10 of the p-value from tumor versus control miRNA expression counts. Labelled miRNAs have Log2 fold change greater than 2SD from the mean.

A total of fifteen samples from enucleated specimens (7 medulloepitheliomas and 8 controls) were analyzed with the Affymetrix GeneChip miRNA Arrays. Many miRNAs were differentially expressed in the two groups as highlighted in the volcano plot in Fig. 1. MiRNAs that were over- or underexpressed by at least 2 standard deviations with a significant p-value (p < 0.05) are listed in Table 2 and Table 3, respectively.

**Table 2:** MiRNAs significantly overexpressed (p < 0.05) by at least 2 standard deviations in tumors compared to control samples.

*MiRNA Symbol*	*ME vs*. *CT 15R (p-value)*	*ME vs*. *CT 15R Lin(FC)*	*ME vs*. *CT 15R Log2(FC)*	*ME vs*. *CT Standard deviation*
miR-4513	0.00083826	1.82565	0.868414	+2s
miR-331-5p	0.00107521	2.30358	1.20388	+2s
miR-1296	0.00400858	2.0393	1.02807	+2s
miR-4454	0.00506333	2.33973	1.22634	+2s
miR-181c-star	0.00864938	2.72817	1.44793	+2s
miR-3651	0.0104184	2.29852	1.2007	+2s
miR-654-5p	0.0144585	1.9331	0.950914	+2s
miR-550a	0.0155852	2.30192	1.20284	+2s
miR-324-5p	0.0156695	2.37185	1.24601	+2s
miR-99b-star	0.0203453	2.58034	1.36756	+2s
miR-4526	0.0254955	1.8913	0.919377	+2s
miR-4667-5p	0.0256011	1.90105	0.926796	+2s
miR-542-5p	0.0291441	2.25941	1.17595	+2s
miR-3175	0.030082	2.92621	1.54903	+2s
miR-151-3p	0.0310334	1.98682	0.99046	+2s
miR-452	0.0313143	2.47711	1.30866	+2s
miR-3065-5p	0.0315675	2.14354	1.09999	+2s
miR-887	0.0351422	1.89504	0.922229	+2s
miR-150-star	0.0386284	2.08094	1.05724	+2s
miR-629-star	0.0390014	2.00566	1.00408	+2s
miR-454	0.0394097	2.10381	1.073	+2s
miR-431	0.040385	2.88151	1.52683	+2s
miR-769-3p	0.0415066	1.83048	0.872224	+2s
miR-299-3p	0.0415905	1.90425	0.929225	+2s
miR-550a-star	0.0423132	2.12592	1.08809	+2s
miR-1287	0.0423992	1.91762	0.939317	+2s
miR-4783-3p	0.0452132	2.05298	1.03772	+2s
miR-337-5p	0.045312	2.05673	1.04036	+2s
miR-181a	0.045623	2.53466	1.34179	+2s
miR-296-3p	0.0465991	2.09545	1.06726	+2s
miR-489	0.0476999	2.73638	1.45227	+2s
miR-628-3p	0.0487057	2.37865	1.25014	+2s
miR-331-3p	0.00072146	4.77257	2.25477	+3s
miR-200c	0.00171416	3.74303	1.90421	+3s
miR-25	0.00228449	3.99073	1.99665	+3s
miR-483-5p	0.00374816	3.16618	1.66274	+3s
miR-210	0.0037929	4.45366	2.15499	+3s
miR-106b-star	0.00534338	3.76198	1.91149	+3s
miR-92a	0.00831397	2.99751	1.58377	+3s
miR-34a	0.009654	4.68914	2.22932	+3s
miR-19a	0.0105539	3.11789	1.64057	+3s
miR-425	0.0119682	3.46944	1.7947	+3s
miR-92a-1-star	0.0157259	3.31281	1.72806	+3s
miR-130a	0.0168223	3.05017	1.60889	+3s
miR-92b	0.0196283	4.24878	2.08705	+3s
miR-181a-star	0.0196711	4.49802	2.16929	+3s
miR-18a-star	0.0198721	3.17976	1.66892	+3s
miR-375	0.0297981	3.74662	1.90559	+3s
miR-370	0.0324271	3.04826	1.60799	+3s
miR-149	0.0341333	3.5908	1.8443	+3s
miR-629	0.0395607	4.6143	2.20611	+3s
miR-345	0.0412484	3.62454	1.8578	+3s
miR-376c	0.0428205	3.40819	1.76901	+3s
miR-135a-star	0.00010313	7.59616	2.92527	+6s
miR-769-5p	0.00019315	5.43666	2.44272	+6s
miR-4417	0.00023069	14.204	3.82823	+6s
miR-21	0.00031135	5.50629	2.46108	+6s
miR-20a	0.00034234	9.30564	3.21811	+6s
miR-106b	0.0004506	7.6127	2.92841	+6s
miR-106a	0.00053755	7.16934	2.84184	+6s
miR-17	0.00055035	7.07773	2.82329	+6s
miR-18b	0.00086543	10.1821	3.34796	+6s
miR-25-star	0.00097707	5.66118	2.5011	+6s
miR-20b	0.00101489	7.14623	2.83718	+6s
miR-93	0.00147512	5.09062	2.34784	+6s
miR-3687	0.00153801	7.4984	2.90658	+6s
miR-551b	0.00171105	5.46106	2.44918	+6s
miR-17-star	0.00202618	7.55628	2.91768	+6s
miR-19b	0.00440786	7.2896	2.86584	+6s
miR-7	0.00478394	12.5187	3.64601	+6s
miR-421	0.00618539	5.00526	2.32344	+6s
miR-93-star	0.0099438	5.25781	2.39446	+6s
miR-487a	0.011085	4.93567	2.30324	+6s
miR-15a	0.0139553	6.01482	2.58852	+6s
miR-363	0.0148836	6.16909	2.62506	+6s
miR-9-star	0.0166476	6.34056	2.66461	+6s
miR-382	0.0235705	5.15825	2.36688	+6s
miR-124-star	0.0273617	5.86526	2.5522	+6s
miR-891a	0.0330171	5.4209	2.43853	+6s
miR-487b	0.0383924	5.56007	2.4751	+6s
miR-182	0.0453814	15.8393	3.98544	+6s
miR-217	1.68E-05	166.311	7.37774	> +6s
miR-216a	3.53E-05	73.8658	6.20683	> +6s
miR-130b	7.78E-05	40.908	5.35431	> +6s
miR-216b	7.79E-05	71.4398	6.15866	> +6s
miR-18a	9.16E-05	30.4575	4.92873	> +6s
miR-184	0.00057019	28.0727	4.81109	> +6s
miR-124	0.00224884	54.4488	5.76683	> +6s

**Table 3:** MiRNAs significantly underexpressed (p < 0.05) by at least 2 standard deviations in tumors compared to control samples.

*MiRNA Symbol*	*ME vs*. *CT 15R (p-value)*	*ME vs*. *CT 15R Lin(FC)*	*ME vs*. *CT 15R Log2(FC)*	*ME vs*. *CT Standard deviation*
miR-4298	7.31E-05	-1.95551	-0.967547	-2s
let-7c	0.00038689	-1.91379	-0.936431	-2s
miR-574-5p	0.00404375	-1.82692	-0.86941	-2s
miR-509-3-5p	0.00509148	-1.841	-0.88049	-2s
miR-508-5p	0.0101842	-1.84679	-0.885021	-2s
miR-3921	0.01021	-1.7505	-0.807766	-2s
miR-30a-star	0.0113581	-2.45667	-1.2967	-2s
let-7d	0.0131867	-2.08921	-1.06296	-2s
miR-99a	0.0133064	-1.97959	-0.985198	-2s
miR-126	0.0142176	-1.75444	-0.811007	-2s
miR-3135b	0.0155168	-1.72398	-0.785747	-2s
miR-3609	0.0171527	-1.83873	-0.878707	-2s
miR-23a	0.0186938	-1.69122	-0.758066	-2s
miR-155	0.0217847	-1.63886	-0.712694	-2s
miR-3613-3p	0.0246578	-1.54502	-0.627627	-2s
miR-125b-2-star	0.0260984	-2.33265	-1.22197	-2s
miR-1184	0.0261281	-1.7633	-0.818276	-2s
miR-4329	0.0265162	-1.89642	-0.92328	-2s
miR-1207-5p	0.0270965	-1.52179	-0.605774	-2s
miR-455-3p	0.0363036	-1.66499	-0.735512	-2s
miR-4487	0.0397386	-1.54414	-0.626808	-2s
miR-4701-3p	0.044214	-1.67333	-0.742725	-2s
let-7b	0.00026306	-2.63302	-1.39672	-3s
miR-150	0.00080125	-3.03867	-1.60344	-3s
miR-328	0.0014731	-2.59841	-1.37763	-3s
miR-193a-5p	0.00215253	-3.28019	-1.71378	-3s
miR-214	0.00347545	-3.73469	-1.90099	-3s
miR-574-3p	0.00654517	-3.28345	-1.71521	-3s
miR-532-3p	0.0107958	-2.70874	-1.43762	-3s
miR-422a	0.0125962	-3.28801	-1.71722	-3s
miR-204	0.0129536	-3.31134	-1.72742	-3s
miR-378	0.020049	-2.93103	-1.55141	-3s
miR-1973	0.0215745	-2.51265	-1.32921	-3s
miR-378f	0.0268653	-3.42704	-1.77696	-3s
miR-133b	0.0396013	-3.55802	-1.83107	-3s
miR-1911	0.0473435	-3.19215	-1.67453	-3s
miR-509-3p	8.02E-06	-14.603	-3.8682	-6s
miR-146a	4.04E-05	-9.91478	-3.30958	-6s
miR-145	0.00090581	-4.41564	-2.14262	-6s
miR-29b-2-star	0.0034838	-5.02666	-2.3296	-6s
miR-187	0.010088	-4.60929	-2.20455	-6s
miR-133a	0.044119	-4.99992	-2.3219	-6s
miR-211	2.46E-07	-42.9191	-5.42355	-6s

Seven of these miRNAs–miR-124, miR-130b, miR-18a, miR-184, miR-217, miR-216a, miR-216b –were overexpressed by more than 6 standard deviations in tumors when compared to controls. Seven of these miRNAs–miR-509-3p, miR-146a, miR-145, miR-29b-2-star, miR-187, miR-133a, miR-211–were underexpressed in tumors versus controls by at least 6 standard deviations from the mean.

Some tumors included neuronal and glial type tissue as well as cartilage in one sample. Therefore, we compared the miRNAs that were significantly different among tumor versus control tissues to miRNA profiles described in retinoblastoma, glioblastoma, and cartilage. These comparisons are presented in Table 4. Since there was only one histologically benign medulloepithelioma in our samples, we were unable to compare miRNA differential profiling between malignant and benign tumors. Since the sample size of teratoid and non-teratoid tumors was small, we did not compare miRNA expression differences between these two groups.

**Table 4:** MiRNAs differentially regulated in medulloepitheliomas (n = 7), retinoblastoma, glioblastoma, and chondrocytes (precursor, normal, reactive).

MiRNA	ME vs. CT (±2SD)	Retinoblastoma [46]	Glioblastoma [51]	Chondrocytes (P, N, and R)^†^ [52]
miR-19b	✓		✓	✓ (All)
miR-92a	✓		✓	✓ (All)
miR-214	✓			✓ (All)
miR-382	✓	✓		✓ (P)
miR-106a	✓	✓		✓ (All)
miR-106b	✓	✓	✓	
miR-130a	✓		✓	✓ (P, N)
miR-20a	✓		✓	✓ (All)
miR-574-3p	✓		✓	✓ (All)
miR-455-3p	✓		✓	✓ (All)
miR-210	✓			✓ (All)
miR-370	✓	✓		✓ (N,R)
miR-17	✓	✓	✓	✓ (N,R)
miR-133a	✓			✓ (R)
Let-7b	✓	✓	✓	
Let-7c	✓	✓	✓	
Let-7d	✓		✓	
miR-181a	✓	✓	✓	
miR-193a-5p	✓	✓		
miR-155	✓	✓		
miR-216a	✓	✓		
miR-217	✓	✓		
miR-20b	✓	✓	✓	
miR-25	✓	✓	✓	
miR-93	✓	✓	✓	
miR-34a	✓	✓		
miR-18a	✓	✓		
miR-151-3p	✓		✓	
miR-15a	✓		✓	
miR-19a	✓		✓	
miR-21	✓		✓	
miR-23a	✓		✓	
miR-331-3p	✓		✓	
miR-99a	✓		✓	

^†^ P: Precursor chondrocytes, N: Normal chondrocytes, R: Reactive chondrocytes, All: All three types of chondrocytes (P, N, and R).

### RT-PCR validation

For validation using RT-PCR, we chose three miRNAs (miR-217, miR-216a, and miR-216b) that were upregulated and three miRNAs (miR-146a, miR-509-3p, miR-211) that were downregulated by at least 6 standard deviations in tumor compared to control arrays. After normalization with U6 snRNA as the endogenous control, the RT-PCR data confirmed significant expression (P < 0.05) of all six miRNAs in all samples used in microarray analysis (Table 5).

**Table 5:** qPCR validation Data.

Sample	Control			Medulloepithelioma			Mann Whitney U
	N	Mean Fold-change	Std. Dev. (Range)	N	Mean Fold-change	Std. Dev. (Range)	P-value
**miR-217**	8	0.304	0.450 (0.053–1.38)	7	13.529	13.460 (0.85–38.87)	0.002
**miR-216a**	8	0.312	0.435 (0.015–1.28)	7	23.055	22.109 (1.26–71.90)	0.002
**miR-216b**	8	0.633	1.235 (0.012–3.58)	7	22.614	24.237 (1.20–60.49)	0.002
**miR-146a**	8	2.427	1.834 (0.58–3.79)	7	0.525	0.209 (0.18–0.62)	0.005
**miR-509-3p**	8	4.557	3.214 (0.99–10.82)	7	0.311	0.203 (0.039–0.63)	0.001
**miR-211**	8	2.089	1.788 (0.41–3.67)	7	0.652	0.306 (0.35–1.25)	0.032

### Pathway analysis

Following the addition of upregulated miRNAs, the DIANA-miRPath v2.0 identified 31 pathways (see S1 Table) as significantly enriched (p < 0.05). Among these, the top 15 pathways included those involved in prion disease, infections (Hepatitis B, HTLV-1), PI3-AKT signaling pathway, viral carcinogenesis, and specific type of cancers including colorectal cancer, pancreatic cancer and glioma (see Table 6). Similarly, with the downregulated miRNAs, significant enrichment was seen in 45 pathways that were mainly influenced by 3 miRNAs, namely miR-133b, miR-145-5p, and miR-146a-5p (see S2 Table). In addition to pathways in cancer and different infections, TLR signaling pathways (p < 4.36E-16) and NF-κB signaling pathway (p < 9.00E-06) were among the top 5 associated pathways (Table 7).

**Table 6:** Top 15 pathways significantly influenced by upregulated miRNAs.

KEGG pathway	p-value	#genes^†^	#miRNAs
Prion diseases	0	1	1
Colorectal cancer	0	11	8
Pancreatic cancer	0	13	12
Glioma	0	10	11
Chronic myeloid leukemia	0	24	11
Melanoma	0	20	12
Bladder cancer	0	17	12
Pathways in cancer	0	70	13
Prostate cancer	0	28	13
Hepatitis B	0	41	16
HTLV-I infection	2.22E-15	53	11
Small cell lung cancer	2.88E-13	26	9
Non-small cell lung cancer	2.95E-11	18	14
PI3K-Akt signaling pathway	3.20E-11	23	6
Viral carcinogenesis	2.83E-09	18	8

^†^ Number of genes predicted by TarBase v6.0.

**Table 7:** Top 15 pathways significantly influenced by downregulated miRNAs.

KEGG pathway	p-value	#genes^†^	#miRNAs
Toll-like receptor signaling pathway	4.36E-16	14	3
Hepatitis B	4.50E-10	14	3
Pathways in cancer	8.01E-07	20	3
Pancreatic cancer	7.90E-06	8	3
NF-kappa B signaling pathway	9.00E-06	7	2
Apoptosis	9.00E-06	9	3
Chagas disease (American trypanosomiasis)	1.52E-05	9	3
Tuberculosis	1.52E-05	13	3
Hepatitis C	1.52E-05	10	3
Bladder cancer	1.52E-05	6	3
RIG-I-like receptor signaling pathway	0.000161	7	2
Legionellosis	0.000229	6	3
Pertussis	0.000293	7	3
Measles	0.000293	9	3
Neurotrophin signaling pathway	0.000611	8	3

^†^ Number of genes predicted by TarBase v6.0.

## Discussion

During tumorigenesis, it is suggested that dysregulations of miRNA-mediated gene regulatory networks, evident in many cancer models, play several roles. At the cellular level, miRNAs function as master regulators and signal modulators. They fine-tune gene expression in many complex pathways. When these pathways are disrupted, the resulting alterations permit tumorigenesis within a particular tissue [28]. This study highlights significant changes in the miRNA expression profile in intraocular medulloepitheliomas as compared to controls.

Many of the over- or underexpressed miRNA in medulloepithelioma were reported to play various roles in carcinogenesis. In this section of the discussion, we chose to highlight the function(s) of some of the validated miRNAs that showed expression differences by several standard deviations from the control. In this discussion we will focus on a few of the MiR-217 was highly overexpressed in medulloepitheliomas. Recent data suggests that miR-217 is an oncogene that is overexpressed in aggressive human B cell lymphomas [30] and contradictarorily functions as a potential tumor suppressor in hepatocellular carcinoma through direct suppression of E2F3 [31]. On the other hand, levels of miR-217 are downregulated in pancreatic intraepithelial neoplasm and pancreatic ductal adenocarcinomas [32] and in clear cell renal carcinomas [33]. Further studies are needed to determine whether higher expression of miR-217 in intraocular medulloepithelioma represents an oncogenic effect or the dysregulation functions as a potential tumor suppressor that might explain its low metastatic potential.

MiR-18a was also highly expressed in intraocular medulloepithelioma samples. Recently in an orthotopic metastatic breast cancer xenograft model, miR-18a suppressed distant metastasis via the hypoxia-inducible factor 1-alpha pathway [34]. This observation is interesting since it is well known that intraocular medulloepitheliomas typically spread locally and distant metastasis is rare [14]. It is possible that the overexpression of miR-18a may play a role in repressing the metastatic behavior of intraocular medulloepitheliomas. Additionally, miR-216a and miR-216b in our study following qPCR validation demonstrated a twenty fold change in intraocular medulloepithelioma as compared to the controls. In contrast, both these miRNAs are downregulated in a mouse model of pancreatic cancer [35]. However, overexpression of miR-216a was shown to activate the PI3K/Akt and TGF-β pathways by targeting PTEN and SMAD7, contributing to hepatocarcinogenesis and tumor recurrence in heptaocellular carcinoma. The exact overexpression of these two miRNAs in medulloepithelioma needs further investigation [36]. Interestingly miR-382 was significantly upregulated in ocular medulloepitheliomas in this study. This miRNA functions as an oncogene under hypoxic conditions and also regulates tumorigenesis through the PTEN/AKT/mTOR pathway [37].

Three miRNAs (miR-146a, miR-509-3p and miR-211) showed significantly reduced expression in the intraocular medulloepithelioma specimens. Downregulation of miR-146a was shown to play a role in migration and metastasis of breast carcinoma [38] and miR-509-3p was shown to be a tumor suppressor in renal cell carcinoma with downregulation associated with cell invasion and migration in renal cell cancer [39]. MiR-211 downregulation has been shown in a variety of cancers and is associated with tumor progression invasion in breast carcinoma, tumors of the head and neck and melanomas [38, 40, 41]. Interestingly miR-211 is found in abundance in the vitreous humor and one might suggest that the source of this miRNA may potentially be the ciliary epithelium or the sensory retina [21].

Though a panel of six miRNAs was highly over- or underexpressed and validated in the medulloepithelioma specimens, none of them were unique to the tumor. It is possible that a combination of this panel might be useful as biomarkers for medulloepitheliomas for undifferentiated neuronal tumors of the eye and adnexa. Both miR-216a and miR-216b are associated with various types of cancer in particular adenocarcinoma of the pancreas. MiR-146a is believed to be involved in the regulation of inflammation and major pathways in cancer. As for miR-217, there is no specific pathway identified linked to this microRNA, but is indirectly associated with cancer and pancreatitis. Furthermore, miR-146a is directly involved in various types of cancer including prostate, gastric, sarcoma, leukemia and pancreatic cancer; and miR-509-3p is involved in renal cell carcinoma with no direct link to a specific pathway. Finally, miR-211 is associated with cancer and stroke. So clearly, most of our differentially expressed miRNAs are related to cancer and signaling pathways (see Tables 6 and 7).

Ocular medulloepithelioma has been associated with a DICER1 germline mutations in familial pleuropulmonary blastoma (PPB) by genome-wide linkage analysis [42]. DICER1 is located on chromosome 14q32 and encodes a ribonuclease that participates in miRNA formation [43]. In this series, the ocular medulloepithelioma are localized in all patients and did not show the phenotype typical of DICER mutations [42]. Furthermore dicer knockouts typically result in a global depression in miRNA production [44]. In a patient with DICER mutation and mutated PPB elevated serum levels of miR-125a-3p and miR-125b-2-3p were described. These miRNAs were not upregulated in the tumor samples in this study [45].

Retinoblastoma like ocular medulloepthelioma is another ocular neoplasm that is common among children. Though the clinical features and behavior of retinoblastoma is quite different from medulloepithelioma, they share some common pathologic features that include the undifferentiated primitive neuronal cells and presence of rosettes in the tumor. Our study detected many significantly up- and downregulated miRNAs in the tumor tissue of medulloepithelioma that were also described in retinoblastoma as noted in Table 4. Of particular interest is the significant downregulation of the let-7 family of miRNAs described in retinoblastoma [46] and also seen in the intraocular medulloepitheliomas in our study. The let-7 miRNAs are a family of seven subtypes that play an important role in the development and differentiation of embryonic cells into specific lineage in the central nervous system. In addition many members of the let-7 family play a role in cancer as a tumor suppressing miRNA [47] but interacts with LIN28, which encodes an RNA binding protein, as is described later in this discussion.

Medulloepitheliomas are primitive neuroepithelial tumors that pathologically resemble primitive neuroectodermal tumors of the central nervous system [48]. Detailed miRNA profiling in primitive neuroectodermal tumors is currently unavailable. In cell lines of primitive neuroectodermal tumors, miR-125 was upregulated in response to chemotherapy [49]. Significant dysregulation of miR-125 was not seen in our tumor samples.

Embryonal tumor with multilayered rosettes (ETMR) is an aggressive primitive neuroectodermal neoplasm of the central nervous system. In the central nervous system, abundant neutrophils and true rosettes in the embryonal tumors suggest that ependymoblastoma and medulloepithelioma are the same entity [50]. Intraocular medulloepitheliomas share some of the histological features of these tumors; however, the result is rarely metastasis or mortality, even in the malignant histological variety [11]. LIN28, an RNA binding protein, is an important diagnostic marker, which is upregulated in many cancers including ETMR. In an ETMR cell line, LIN28 knockdown showed an increase in let-7 expression by activating downstream pathways. It is suggested that the LIN28/let-7 pathway plays a critical role in the pathophysiology of malignant neoplasms such as ETMR and germ cell tumors. Overexpression of LIN28 selectively inhibits let-7 biogenesis leading to decreased expression of let-7 in such neoplasms. It is possible that the significant decrease in let-7 expression seen in intraocular medulloepitheliomas plays a significant role in the pathogenesis of this neoplasm. The less aggressive behavior of intraocular medulloepitheliomas, in contrast to those seen in the central nervous system, are likely regulated by other mechanisms including some of the miRNAs described earlier in the discussion [47].

Medulloepitheliomas may either be benign or malignant neoplasms [13]. In this series, six tumors were classified as malignant and one as benign. The miRNA differences between malignant versus benign samples could not be studied in view of the disparate numbers. In order to identify specific miRNAs that might be dysregulated in tumors versus controls, the data was analyzed. As shown in Table 4, several miRNAs showed significant differential expression. We also compared this dysregulation to published reports on miRNA alterations in retinoblastoma, glioblastoma, and in embryonic, normal and hypertrophic cartilage [46, 51, 52]. Of the total thirty-four miRNAs that were significantly dysregulated in medulloepithelioma, twenty-two were reported to be dysregulated in malignant glioblastomas [51] suggesting common molecular threads between these primitive neoplasms.

Furthermore, thirteen miRNAs that were dysregulated in intraocular medulloepitheliomas were also expressed in normal, embryonic and/or hypertrophic cartilage. Based on this analysis, the role of these miRNAs, if any, in the formation of cartilaginous medulloepitheliomas needs to be further investigated.

Several pathways appear to be enriched by the upregulated miRNAs. Among the top 15 pathways, the pathway most significantly was associated with prion disease (p < 1e-16) was found to be influenced by a single miRNA, miR-130b-3p, which was predicted to target a single transcript *PRNP* (prion protein gene). Mutations in *PRNP* are associated with human prion diseases [53]. The functional role of this pathway in medulloepithelioma is unclear, thus needs further investigation. Other pathways that may be of interest include transforming-growth-factor-beta (TGF-β) signaling pathway, which was most significantly influenced by miR-20a-5p (p < 9.6e-08) and miR-17-5p (p < 2.7e-07), and hypoxia-inducible factor (HIF)-1 signaling pathway, which was most significantly influenced by miR-20b-5p (p < 4.3e-07) (see S1 Table for the complete list). Upregulation of HIF1-alpha (HIF-1α) is associated with adverse outcomes in patients with neuroblastoma [54] and other tumors as previously described in this report.

Similarly, 45 pathways corresponding to the downregulated miRNAs were noted (see S2 Table). These pathways were mainly influenced by miR-133b, miR-145-5p, and miR-146a-5p. Besides pathways in cancer and different infections, TLR (p<4.36E-16) and NF-κB-signaling pathways (p < 9.00E-06) were among the top 5 associated pathways.

Most cancers are characterized by activation of the NF-κB pathway, resulting in cancer cell proliferation, survival, angiogenesis and metastasis. It appears that tumorigenesis in intraocular medulloepitheliomas may also be influenced by the NF-κB pathway [55].

TLR stimulation can lead to up- or downregulation of various miRNA expressions. TLRs are known to trigger innate immune system and bolster adaptive immunity against antigens expressed by pathogens and tumor cells [56], and can also modulate anti-cancer therapy [57]. Patients with breast cancer carry the loss-of function allele for *TLR4*. The defective allele affects the binding of high-mobility group box (HMGB1) protein to TLR4. Patients with the loss-of-function TLR4 allele have been shown to relapse more quickly after radiotherapy or chemotherapy indicating the clinical relevance of immunoadjuvant pathway triggered by tumor cell death [58, 59]. MiR-146a, which was significantly downregulated in intraocular medulloepitheliomas in this study, may interact with both the NF-κB pathway as well as the TLR pathway as shown in breast and thyroid cancer [60]. Studies have shown the pathological relevance of NF-κB/miR-146 in human breast cancer, pancreatic cancer, anaplastic thyroid carcinomas, and brain tumors [61]; therefore, one might suggest that this pathway may play a role in the pathogeneis of medulloepitheliomas. In the CNS gliomas, TLR pathways are responsible for converting microglia into a glioma supportive phenotype [62]. Such a role in medulloepitheliomas needs further investigation.

There are some limitations of this particular study. The sample size of the study was small making it difficult to analyze miRNA alterations in subsets of medulloepitheliomas. More specimens are needed to perform additional analysis. Also, intraocular medulloepitheliomas are localized tumors and determining mortality based prognostic factors based on miRNA expression may not be relevant. We hope that future studies will be able to address the differences in the biological behavior of central nervous system medulloepitheliomas and intraocular medulloepitheliomas at the molecular level.

## Supporting Information

S1 Table DIANA-miRPath v2.0 identified significantly enriched pathways for upregulated miRNAs. (DOCX)

S2 Table DIANA-miRPath v2.0 identified significantly enriched pathways for downregulated miRNAs. (DOCX)
