# Supplementary material for: MicroRNA Profiling in Intraocular Medulloepitheliomas
Source: PLoS One. 2015 Mar 25;10(3):e0121706. doi: 10.1371/journal.pone.0121706 (PMC4373864; doi:10.1371/journal.pone.0121706)
Supplement: S1 Table — (DOCX) [file pone.0121706.s001.docx]

**Table S1:** DIANA-miRPath v2.0 identified significantly enriched pathways for up-regulated miRNAs.

| **KEGG pathway** | **p-value** | **#genes** | **#miRNAs** |
| --- | --- | --- | --- |
| Prion diseases | 0 | 1 | 1 |
| Colorectal cancer | 0 | 11 | 8 |
| Pancreatic cancer | 0 | 13 | 12 |
| Glioma | 0 | 10 | 11 |
| Chronic myeloid leukemia | 0 | 24 | 11 |
| Melanoma | 0 | 20 | 12 |
| Bladder cancer | 0 | 17 | 12 |
| Pathways in cancer | 0 | 70 | 13 |
| Prostate cancer | 0 | 28 | 13 |
| Hepatitis B | 0 | 41 | 16 |
| HTLV-I infection | 2.22E-15 | 53 | 11 |
| Small cell lung cancer | 2.88E-13 | 26 | 9 |
| Non-small cell lung cancer | 2.95E-11 | 18 | 14 |
| PI3K-Akt signaling pathway | 3.20E-11 | 23 | 6 |
| Viral carcinogenesis | 2.83E-09 | 18 | 8 |
| Cell cycle | 4.02E-09 | 29 | 10 |
| Endometrial cancer | 9.49E-08 | 14 | 8 |
| Acute myeloid leukemia | 2.26E-07 | 18 | 8 |
| TGF-beta signaling pathway | 4.01E-06 | 19 | 6 |
| Transcriptional misregulation in cancer | 5.42E-06 | 14 | 8 |
| p53 signaling pathway | 9.63E-06 | 8 | 7 |
| HIF-1 signaling pathway | 6.23E-05 | 6 | 6 |
| Hepatitis C | 7.72E-05 | 29 | 6 |
| Renal cell carcinoma | 0.001471 | 5 | 4 |
| Focal adhesion | 0.002323 | 32 | 6 |
| Epstein-Barr virus infection | 0.003873 | 13 | 6 |
| Glycosaminoglycan biosynthesis - chondroitin sulfate | 0.007903 | 4 | 1 |
| Thyroid cancer | 0.010042 | 6 | 6 |
| ErbB signaling pathway | 0.010397 | 16 | 4 |
| mTOR signaling pathway | 0.011079 | 3 | 4 |
| VEGF signaling pathway | 0.024568 | 15 | 4 |
| Measles | 0.068133 | 11 | 3 |
| Fatty acid elongation | 0.096564 | 6 | 1 |
| Toxoplasmosis | 0.172572 | 23 | 5 |
| B cell receptor signaling pathway | 0.174819 | 6 | 3 |
| Progesterone-mediated oocyte maturation | 0.222301 | 17 | 1 |
| Herpes simplex infection | 0.317898 | 11 | 4 |
| Wnt signaling pathway | 0.340238 | 8 | 3 |
| Insulin signaling pathway | 0.350084 | 8 | 4 |
| Endocytosis | 0.351602 | 13 | 4 |
| Osteoclast differentiation | 0.373191 | 8 | 4 |
| Neurotrophin signaling pathway | 0.431142 | 20 | 2 |
| Adipocytokine signaling pathway | 0.704691 | 1 | 1 |
| MAPK signaling pathway | 0.737876 | 1 | 1 |
| Fatty acid metabolism | 0.75925 | 9 | 1 |
| T cell receptor signaling pathway | 0.7797 | 5 | 3 |
| Influenza A | 0.813065 | 5 | 3 |
| Phosphatidylinositol signaling system | 0.84776 | 1 | 2 |
| Jak-STAT signaling pathway | 0.856815 | 4 | 2 |
| Tight junction | 0.90979 | 2 | 2 |
| Rheumatoid arthritis | 0.911044 | 2 | 2 |
| Apoptosis | 0.914372 | 3 | 1 |
| Adherens junction | 0.941269 | 15 | 2 |
| Endocrine and other factor-regulated calcium reabsorption | 0.941933 | 1 | 1 |
| Tuberculosis | 0.950009 | 1 | 1 |
| GnRH signaling pathway | 0.964068 | 14 | 1 |
| Fc epsilon RI signaling pathway | 0.968218 | 10 | 1 |
| Chagas disease (American trypanosomiasis) | 0.975783 | 3 | 1 |
| Vasopressin-regulated water reabsorption | 0.97646 | 6 | 2 |
| Chemokine signaling pathway | 0.982372 | 1 | 1 |
| NOD-like receptor signaling pathway | 0.984084 | 13 | 2 |
| Huntington's disease | 0.987469 | 1 | 1 |
| Toll-like receptor signaling pathway | 0.988231 | 5 | 2 |
| Shigellosis | 0.990715 | 12 | 2 |
| Circadian rhythm | 0.991189 | 3 | 2 |
| Inositol phosphate metabolism | 0.99213 | 1 | 1 |
| Natural killer cell mediated cytotoxicity | 0.992563 | 1 | 1 |
| Cell adhesion molecules (CAMs) | 0.993364 | 1 | 1 |
| Long-term potentiation | 0.99431 | 1 | 1 |
| Regulation of actin cytoskeleton | 0.994743 | 1 | 1 |
| Cholinergic synapse | 0.995534 | 1 | 1 |
| NF-kappa B signaling pathway | 0.995655 | 4 | 2 |
| Axon guidance | 0.995911 | 18 | 2 |
| Melanogenesis | 0.996039 | 3 | 1 |
| Pathogenic Escherichia coli infection | 0.997809 | 12 | 2 |
| Leishmaniasis | 0.998154 | 3 | 2 |
| Dopaminergic synapse | 0.998583 | 1 | 1 |
| Amphetamine addiction | 0.99869 | 2 | 2 |
| Spliceosome | 0.999018 | 6 | 2 |
| Salmonella infection | 0.999284 | 15 | 1 |
| Gap junction | 0.999371 | 14 | 1 |
| Cytokine-cytokine receptor interaction | 0.99966 | 5 | 1 |
| ECM-receptor interaction | 0.999689 | 11 | 2 |
| mRNA surveillance pathway | 0.999691 | 5 | 2 |
| Notch signaling pathway | 0.999791 | 3 | 2 |
| Epithelial cell signaling in Helicobacter pylori infection | 0.999809 | 12 | 2 |
| Fc gamma R-mediated phagocytosis | 0.999817 | 2 | 2 |
| Dorso-ventral axis formation | 0.999851 | 1 | 2 |
| Malaria | 0.999885 | 1 | 1 |
| Amyotrophic lateral sclerosis (ALS) | 0.99991 | 1 | 1 |
| RNA degradation | 0.99991 | 4 | 1 |
| Viral myocarditis | 0.999917 | 3 | 2 |
| Hedgehog signaling pathway | 0.999926 | 2 | 1 |
| Oocyte meiosis | 0.999948 | 4 | 2 |
| Aldosterone-regulated sodium reabsorption | 0.999951 | 2 | 3 |
| Bacterial invasion of epithelial cells | 0.999961 | 14 | 1 |
| African trypanosomiasis | 0.999969 | 2 | 2 |
| Steroid biosynthesis | 0.99997 | 4 | 1 |
| RIG-I-like receptor signaling pathway | 0.999982 | 2 | 1 |
| Leukocyte transendothelial migration | 0.999993 | 1 | 1 |
| Basal cell carcinoma | 0.999995 | 2 | 2 |
| Amoebiasis | 0.999995 | 14 | 1 |
| Terpenoid backbone biosynthesis | 0.999997 | 5 | 1 |
| Alcoholism | 0.999997 | 1 | 1 |
| Lysine degradation | 0.999998 | 2 | 2 |
| Vascular smooth muscle contraction | 0.999998 | 1 | 1 |
| Staphylococcus aureus infection | 1 | 1 | 1 |
| Pentose and glucuronate interconversions | 1 | 2 | 1 |
| Primary bile acid biosynthesis | 1 | 1 | 1 |
| Pentose phosphate pathway | 1 | 6 | 1 |
| Synthesis and degradation of ketone bodies | 1 | 1 | 1 |
| Valine, leucine and isoleucine degradation | 1 | 8 | 1 |
| Amino sugar and nucleotide sugar metabolism | 1 | 2 | 1 |
| Glycosaminoglycan biosynthesis - keratan sulfate | 1 | 3 | 1 |
| Ether lipid metabolism | 1 | 1 | 1 |
| Sphingolipid metabolism | 1 | 8 | 1 |
| Folate biosynthesis | 1 | 2 | 1 |
| Porphyrin and chlorophyll metabolism | 1 | 2 | 1 |
| PPAR signaling pathway | 1 | 1 | 1 |
| Mismatch repair | 1 | 1 | 1 |
| Homologous recombination | 1 | 1 | 1 |
| SNARE interactions in vesicular transport | 1 | 7 | 1 |
| Phagosome | 1 | 2 | 1 |
| Peroxisome | 1 | 1 | 1 |
| Antigen processing and presentation | 1 | 1 | 1 |
| Cytosolic DNA-sensing pathway | 1 | 2 | 1 |
| Intestinal immune network for IgA production | 1 | 1 | 1 |
| Type II diabetes mellitus | 1 | 1 | 1 |
| Legionellosis | 1 | 2 | 1 |
| Allograft rejection | 1 | 1 | 1 |
| Arrhythmogenic right ventricular cardiomyopathy (ARVC) | 1 | 8 | 1 |
