# Supplementary material for: MicroRNA Profiling in Intraocular Medulloepitheliomas
Source: PLoS One. 2015 Mar 25;10(3):e0121706. doi: 10.1371/journal.pone.0121706 (PMC4373864; doi:10.1371/journal.pone.0121706)
Supplement: S2 Table — (DOCX) [file pone.0121706.s002.docx]

**Table S2:** DIANA-miRPath v2.0 identified significantly enriched pathways for down-regulated miRNAs.

| **KEGG pathway** | **p-value** | **#genes** | **#miRNAs** |
| --- | --- | --- | --- |
| Toll-like receptor signaling pathway | 4.36E-16 | 14 | 3 |
| Hepatitis B | 4.50E-10 | 14 | 3 |
| Pathways in cancer | 8.01E-07 | 20 | 3 |
| Pancreatic cancer | 7.90E-06 | 8 | 3 |
| NF-kappa B signaling pathway | 9.00E-06 | 7 | 2 |
| Apoptosis | 9.00E-06 | 9 | 3 |
| Chagas disease (American trypanosomiasis) | 1.52E-05 | 9 | 3 |
| Tuberculosis | 1.52E-05 | 13 | 3 |
| Hepatitis C | 1.52E-05 | 10 | 3 |
| Bladder cancer | 1.52E-05 | 6 | 3 |
| RIG-I-like receptor signaling pathway | 0.000161 | 7 | 2 |
| Legionellosis | 0.000229 | 6 | 3 |
| Pertussis | 0.000293 | 7 | 3 |
| Measles | 0.000293 | 9 | 3 |
| Neurotrophin signaling pathway | 0.000611 | 8 | 3 |
| Transcriptional misregulation in cancer | 0.000801 | 10 | 3 |
| Influenza A | 0.000836 | 10 | 3 |
| Toxoplasmosis | 0.000931 | 9 | 3 |
| Shigellosis | 0.001206 | 5 | 2 |
| Prostate cancer | 0.001256 | 7 | 3 |
| Rheumatoid arthritis | 0.001891 | 7 | 3 |
| Leishmaniasis | 0.002617 | 6 | 2 |
| Epithelial cell signaling in Helicobacter pylori infection | 0.003556 | 5 | 3 |
| Axon guidance | 0.003979 | 7 | 3 |
| Leukocyte transendothelial migration | 0.004057 | 7 | 3 |
| Cell adhesion molecules (CAMs) | 0.00497 | 5 | 2 |
| Type II diabetes mellitus | 0.005987 | 4 | 2 |
| Osteoclast differentiation | 0.006697 | 7 | 3 |
| Malaria | 0.008103 | 4 | 1 |
| VEGF signaling pathway | 0.008103 | 5 | 2 |
| Amoebiasis | 0.009552 | 6 | 2 |
| Viral myocarditis | 0.011842 | 5 | 2 |
| Colorectal cancer | 0.014141 | 4 | 2 |
| Herpes simplex infection | 0.014252 | 9 | 2 |
| Adherens junction | 0.019574 | 5 | 3 |
| PI3K-Akt signaling pathway | 0.021432 | 14 | 3 |
| Pathogenic Escherichia coli infection | 0.022074 | 4 | 2 |
| Allograft rejection | 0.02345 | 4 | 1 |
| Endometrial cancer | 0.02345 | 4 | 3 |
| T cell receptor signaling pathway | 0.033463 | 6 | 3 |
| Small cell lung cancer | 0.036546 | 5 | 3 |
| Bacterial invasion of epithelial cells | 0.039799 | 4 | 1 |
| Salmonella infection | 0.039799 | 5 | 2 |
| HIF-1 signaling pathway | 0.039799 | 6 | 3 |
| Acute myeloid leukemia | 0.040888 | 4 | 3 |
